# Impact factor 2013 of the Netherlands Heart Journal surpasses 2.0

**DOI:** 10.1007/s12471-014-0538-8

**Published:** 2014-02-27

**Authors:** T. Opthof

**Affiliations:** 1 Department of Clinical and Experimental Cardiology, Heart Failure Research Center, Academic Medical Center, Meibergdreef 9, Room K2-111, 1105 AZ Amsterdam, the Netherlands; 2 Department of Medical Physiology, University Medical Center Utrecht, Utrecht, the Netherlands

**Keywords:** Impact factor, Cardiology

## Abstract

The impact factor of the *Netherlands Heart Journal* was stable at about 1.4 between 2009 and 2012. In 2013 it will break through the 2.0 barrier for the first time.

Having received an impact factor for the very first time in 2009, after many years of hard work from the Editor-in-Chief and his team, the impact factor of the *Netherlands Heart Journal* (NHJ) remained stable at about 1.4 between 2009 and 2012. As anticipated in a previous editorial [1], Fig. 1 shows that it is very highly probable that the impact factor 2013 will break through the 2.0 barrier for the very first time, an increase of over 40 %. ‘Very high probability’ is used here because Thomson Reuters, the company that publishes the impact factor of scientific journals yearly, attributes some of the citations obtained in January and February to the previous year. Thus, some citations obtained in January and February 2013 were attributed to the year 2012 and some obtained in January and February 2014 will be attributed to 2013. Because I have counted from 1 January 2013 till 31 December 2013, this produces an error of about 2–3 %. The estimated impact factor 2013 is at 2.01. At this moment it is difficult to explain this increase. An increase in submissions may have offered the possibility to be more selective in acceptance.

**Fig. 1:**
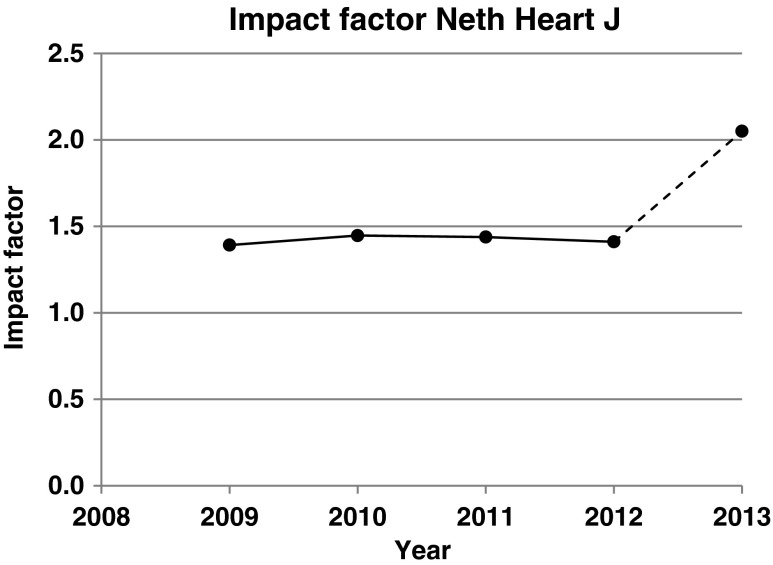
Impact factor of the *Netherlands Heart Journal* between 2009 (first impact factor) and 2013. Values for 2009–2012 are official Thomson Reuters data as published in their *Journal Citation Reports*. The IF 2013 was estimated on the basis of citations in the *Web of Science* between 1 January 2013 and 31 December 2013 to papers published by NHJ in 2011 and 2012

As explained previously [1], the impact factor of 2013 is calculated by summing all citations obtained during 2013 to papers published by NHJ in 2011 or 2012. This total is divided by the total number of articles and reviews published by NHJ in 2011 and 2012. It thus represents the average citation of any article or review published in publication year 2011 or 2012 during citation year 2013. For some papers, e.g. those published in January 2011, the ‘citation window’ is between 24 and 36 months after publication. For others, such as those published in December 2012, the ‘citation window’ is between 1 and 13 months.

The impact factor has often been criticised as a bibliometric parameter because it presents an average of a heavily skewed distribution [2–5]. Despite this, it is still important for authors, editors, readers and publishers and it has survived criticism for about 40 years. In my opinion its use is only acceptable in judging the quality of scientific journals, for which it was developed, not as a tool for decisions on positions or grants of individual scientists [6–8] (but see also Van Kammen et al. [9]).

Figure 2 shows the immediacy index of NHJ over the same years as in Fig. 1. Compared with the impact factor, it is easier to explain and understand. It is simply the average number of citations obtained by all published items *during* the year of publication. Thus, the immediacy index of 2013 is the total number of citations obtained by the NHJ items published in 2013 during the same year, divided by the total number of articles and reviews. Having been at about 0.50 during the last 4 years, it will increase to 1.22 in 2013. It goes without saying that it can be anticipated that a higher immediacy index in 2013 constitutes a solid base for the impact factors to follow in 2014 and 2015.

**Fig. 2:**
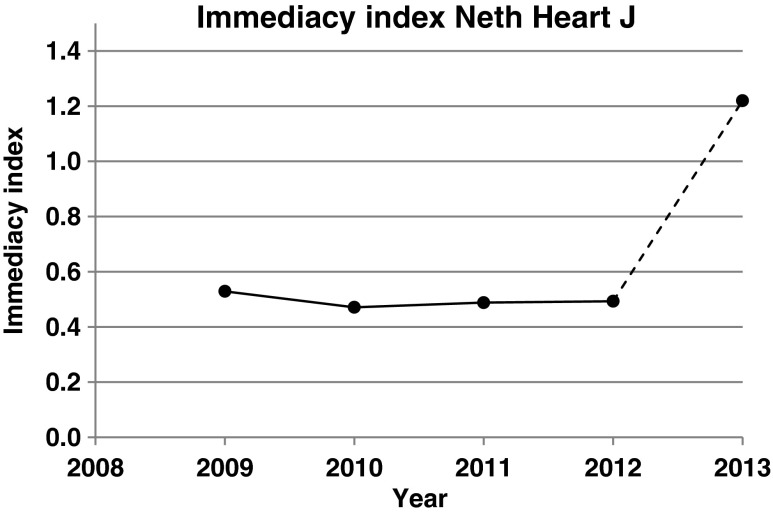
Immediacy index of the *Netherlands Heart Journal* between 2009 (first immediacy index) and 2013. Values for 2009–2012 are official Thomson Reuters data as published in their *Journal Citation Reports*. The immediacy index 2013 was estimated on the basis of citations in the *Web of Science* between 1 January 2013 and 31 December 2013 to papers published by NHJ in 2013

Journals publish more items than original articles and reviews. They may publish editorials, letters, case reports, statements, guidelines etc. Citations to all these items are counted in the numerator of the impact factor. However, the denominator of the impact factor is only determined by the sum of the original articles and reviews published. Citations to items other than these are thus a ‘free lunch’. This makes the impact factor vulnerable to manipulation, as does excessive journal self-citation whether coerced by Editors or not [10, 11]. Figure 3 shows the impact factor of NHJ based on either original articles only, or on reviews only. These impact factors would have been even higher (at 2.21 and 2.19 respectively) than the predicted impact factor of the whole contents. For most cardiovascular journals, reviews are cited at least twice as frequently as original articles. Therefore, it can be stated that the citation of original articles forms the backbone of the impact factor of the *Netherlands Heart Journal* in 2013. This should stimulate more authors to consider NHJ for submission of their work.

**Fig. 3:**
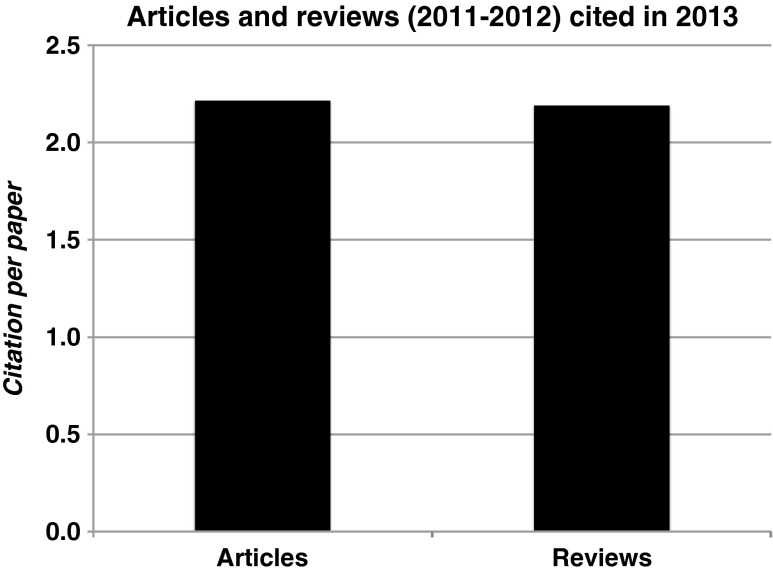
Average citation of articles or reviews published by the *Netherlands Heart Journal* in 2011 and 2012 during 2013. These numbers can be regarded as an impact factor for 2013, if the journal had only published original articles or only reviews
